# Biological clocks and blood clots in space: circadian disruption-induced modulation of thrombosis risk

**DOI:** 10.1016/j.rpth.2026.106840

**Published:** 2026-07-14

**Authors:** Zeinab Ibrahim, Ronan P. Murphy, Germaine Cornelissen Guillaume, David Andrew Green, Roopen Arya, Suzanne C. Cannegieter, Nandu Goswami

**Affiliations:** 1 Center for Spaceflight and Aviation Medicine, Mohammed Bin Rashid University of Medicine and Health Sciences, Dubai, United Arab Emirates; 2 Integrative Cell & Molecular Physiology Group, School of Health & Human Performance, Dublin City University, Glasnevin, Dublin, Ireland; 3 Halberg Chronobiology Center, University of Minnesota, Minneapolis, Minnesota, USA; 4 KBR GmbH, Cologne, Cologne, Germany; 5 Centre of Human and Applied Physiological Sciences, King’s College London, London, UK; 6 Institute for Risk and Disaster Reduction, University College London, London, UK; 7 King’s Thrombosis Centre, Department of Haematological Medicine, King’s College Hospital Foundation National Health Service Trust, London, UK; 8 Department of Clinical Epidemiology, Leiden University Medical Center, Leiden, Netherlands; 9 Gravitational Physiology and Medicine Research Unit, Division of Physiology and Pathophysiology, Otto Loewi Research Center of Vascular Biology, Immunity and Inflammation, Medical University of Graz, Austria

**Keywords:** circadian rhythm, coagulation, hemostasis, endothelial dysfunction, platelet activation, microgravity, spaceflight, space medicine, thrombosis, venous thromboembolism

## Abstract

Long-duration space missions expose astronauts to microgravity, radiation, confinement, fluid redistribution, and circadian disruption, which together induce physiological adaptations, including cardiovascular hemodynamics. Emerging evidence suggests that spaceflight may modulate hemostasis and potentially increase thrombotic risk in the head/neck; however, the association of circadian disruption and hemostasis in space, and its ground-based analogs, has yet to be systematically evaluated. This systematic review aimed to synthesize evidence from real-spaceflight missions and ground-based analogs to (i) characterize thrombosis-related and hemostatic adaptations, (ii) evaluate circadian remodeling under altered gravitational conditions, and (iii) identify mechanistic and methodological gaps linking biological timing to coagulation regulation in space. A systematic search of PubMed, Scopus, and Web of Science was conducted from January 2016 to January 2026. Following Preferred Reporting Items for Systematic Reviews and Meta-Analyses guidelines, studies investigating circadian rhythms, clock-gene regulation, autonomic chronobiology, hemostasis, thrombosis, platelet biology, and coagulation pathways, in spaceflight or spaceflight analogs were systematically screened and analyzed. Thirty-eight studies met inclusion criteria, of which 20 (52.6%) primarily examined hemostasis and thrombosis outcomes and 18 (47.4%) focused on circadian regulation. Thrombosis-related investigations demonstrated dynamic modulation of coagulation cascades, platelet activation pathways, fibrinogen levels, endothelial markers, and complement components across dry immersion, head-down bed rest, animal unloading models, radiation exposure, hypergravity, and real astronaut missions. However, most hemostatic studies relied on end point or milestone-based sampling without circadian-phase resolution. In contrast, circadian-focused studies used dense temporal sampling and revealed phase shifts, altered autonomic rhythmicity, and disruption of molecular clock regulators under simulated and real space conditions, yet rarely assessed direct thrombotic end points. Importantly, no included study simultaneously assessed circadian-phase regulation and hemostatic outcomes, highlighting a critical lack of mechanistic evidence linking biological timing to thrombotic regulation in spaceflight environments. The absence of circadian phase–resolved hemostatic assessment represents a fundamental mechanistic gap. Future integrative chronothrombotic study designs are required to determine whether disrupted biological timing directly contributes to thrombotic vulnerability during spaceflight.

## Essentials

• Blood clotting and cardiovascular events follow strong circadian rhythms.
• Spaceflight disrupts circadian timing and alters coagulation pathways.
• Yet circadian biology and thrombosis are rarely studied together in space.
• Integrating chronobiology with hemostasis may reveal hidden thrombotic risk.

## Graphical abstract

**Figure:**
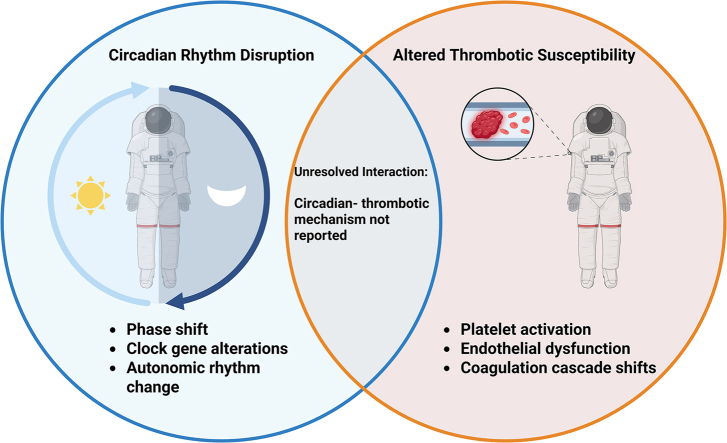

## 1 Introduction

Human spaceflight has evolved from short orbital missions to continuous habitation in Low Earth Orbit (LEO) facilitated by long-duration (≈6 months) missions with career—and thus medically selected—astronauts. While medical events are rare, there are substantial chronic physiological adaptations to microgravity, and acute adaptations to hypergravity exposure during launch and landing [1]. However, with increasing commercial spaceflight in LEO and exploration missions to the Moon and beyond [2], a broader population of individuals will participate in space travel, including those with a greater susceptibility to medical conditions. At the same time, mission architecture imposes constraints on medical care, including limited volume, mass, and power resources, as well as communication delays with ground support [3].

Long-duration space missions expose astronauts to multiple environmental stressors, including altered gravitational loading, cosmic radiation, confinement, fluid shifts, and circadian disruption, which together compromise physiological homeostasis and overall health [4,5]. These changes occur across multiple physiological systems and scales, highlighting the need for integrative approaches to understand complex spaceflight adaptations [6]. An emerging medical concern during long-duration missions is the potential for thrombotic dysregulation. Previous analyses have suggested that thrombotic risk during spaceflight may represent an underrecognized clinical challenge in human space exploration [7]. In 2020, an astronaut who had spent 2 months in LEO aboard the International Space Station (ISS) was diagnosed with obstructive venous thrombosis of the left internal jugular vein, marking the first clinically documented case of spaceflight-associated venous thromboembolism and raising critical questions regarding coagulation control in microgravity [8]. The thrombosis was asymptomatic but was observed as an incidental finding during a scientific study of left internal jugular vein flow [9]. Although identification of a thrombosis appears isolated, the same study reported internal jugular vein flow modulation, including apparent stasis and retrograde flow in several crew members [9], with a relatively high rate in a subsequent medical surveillance study [10].

Furthermore, astronaut and cosmonaut investigations have revealed measurable alterations in coagulation markers, endothelial function, platelet-associated proteins, and inflammatory mediators during or shortly after spaceflight [[11], [12], [13], [14]]. Interestingly, generally less impressive changes in such parameters have been reported in ground-based analogs [15], indicative that unloading per se may not be the only contributor. Given the disparate findings and lack of concurrent measurements, the pathophysiological mechanisms underpinning potential thrombus formation in space are unknown [16].

Importantly, hemostasis on Earth is tightly regulated by the circadian timing system. Endothelial function, platelet reactivity, coagulation cascade activity, and fibrinolysis exhibit diurnal variation driven by clock-controlled gene networks and neuroendocrine signaling [17,18]. Circadian misalignment can disrupt these regulatory pathways, altering platelet activation, coagulation cascade activity, and fibrinolytic balance, thereby influencing temporal susceptibility to thrombotic events [17]. These observations establish biological timing as a fundamental regulator of hemostatic homeostasis and a key determinant of thrombotic risk rather than a secondary modifier of vascular events.

Equally important, the space environment disturbs the circadian rhythm [5]. As an example, astronauts aboard the ISS follow a highly structured 24-hour schedule designed to support circadian alignment. Coordinated Universal Time is used onboard, and astronauts typically awaken at ∼6:00 am and initiate sleep at ∼9:30 pm, with daily activities including work, meals, and exercise scheduled at fixed times [19]. To mitigate the effect of the ∼16 orbital light–dark cycles per day, windows are covered during designated night periods, and artificial lighting is used to simulate a regular diurnal rhythm [19]. These environmental and behavioral cues, including controlled light exposure, scheduled physical activity, and consistent meal timing, act as circadian synchronizers. However, despite these countermeasures, the spaceflight environment characterized by microgravity, confinement, operational demands, and altered light exposure can still disrupt circadian regulation and sleep–wake patterns [19,20]. Circadian rhythms regulate sleep–wake cycles, autonomic function, hormonal secretion, vascular responsiveness, platelet activation, and the rhythmic expression of fundamental clock genes, including *BMAL1*, *CLOCK*, *PER*, *CRY*, and *NR1D1* [21]. Emerging investigations show that circadian rhythm disruption elevates platelet reactivity, fibrinogen concentrations, and thrombotic events, with established morning peaks in myocardial infarction and stroke rates [22]. Spaceflight conditions, including altered light–dark exposure, sleep fragmentation, confinement, and psychosocial stress, are capable of desynchronizing central and peripheral clocks, thereby perturbing neuroendocrine, immune, and vascular pathways that interface directly with hemostatic control [23,24]. Nonetheless, despite the recognized chronobiological impact on thrombosis, spaceflight studies have predominantly investigated coagulation and circadian systems independently.

Recent work has explored the role of circadian clock regulation in hemostasis [25]. Building on these findings, the present companion review specifically evaluates how circadian biology may interact with thrombotic and hemostatic adaptations in spaceflight and spaceflight analog environments. The present systematic review aimed to synthesize evidence across real-spaceflight missions and ground-based analogs to evaluate (i) hemostatic and thrombotic adaptations under space-relevant stressors, (ii) circadian remodeling under altered gravitational and confinement conditions, and (iii) potential mechanistic intersections between biological timing and coagulation regulation. By bridging these domains, we aimed to advance a conceptual framework in which thrombotic risk in space is considered not solely a consequence of mechanical unloading or radiation exposure but also potentially a time-dependent biological process shaped by disrupted circadian control.

## 2 Methods

This systematic review was conducted in accordance with the Preferred Reporting Items for Systematic Reviews and Meta-Analyses guidelines [26], establishing a priori objectives, eligibility criteria, search terms, screening procedures, and methods for data extraction and synthesis.

### 2.1 Eligibility criteria

Studies extracted from PubMed, Scopus, and Web of Science were screened and selected according to predefined inclusion and exclusion criteria (Table 1). Eligible studies including the a priori search terms (Table 2) investigated the effects of spaceflight or spaceflight analog environments—including dry immersion, head-down bed rest, simulated microgravity, hypergravity, or radiation exposure on hemostasis, thrombosis, platelet biology, or circadian regulation in human, animal, or *in vitro* models. Exclusion criteria comprised review articles, conference abstracts, editorials, letters to the editor, book chapters, non-English publications, studies lacking coagulation- or thrombosis-related outcomes, and reports without accessible full text.

**Table 1:** Eligibility criteria for study selection.

Category	Inclusion criteria	Exclusion criteria
Publication year	Studies published between 2016 and 2026	Studies published outside this timeframe
Study type	Primary research studies; includes human, animal, and *in vitro* studies conducted in spaceflight, microgravity, or validated ground-based spaceflight analogs	Reviews, systematic reviews, meta-analyses, editorials, commentaries, opinion papers, and meeting abstracts
Topic relevance	Studies examining either (i) coagulation, thrombosis, platelet biology, or fibrinolysis or (ii) circadian rhythms, circadian disruption, or clock-gene regulation in the context of spaceflight or spaceflight analog exposure	Articles unrelated to space physiology or not addressing circadian or hemostasis-related mechanisms
Intervention/exposure	Spaceflight, microgravity, or ground-based analog exposure with assessment of circadian and/or hemostatic alterations	No spaceflight-related exposure
Outcomes	Assessment of at least 1 hemostatic outcome, including coagulation parameters, thrombotic events, platelet function or activation, thrombus formation, fibrinolysis, or expression of coagulation- or platelet-related genes or proteins, or at least a circadian-related outcome (eg, rhythmicity measures or clock-gene expression)	No circadian- or hemostasis-related outcome measurable or reported
Data quality	Studies with sufficient methodological details and extractable quantitative or qualitative data	Studies with inadequate methodology, insufficient data, or inaccessible full text
Language	English	Non-English publications
Publication type	Peer-reviewed articles	Nonscientific publications, news, websites, or non–peer-reviewed content

**Table 2:** Search keywords.

Arm	Search focus	Keywords used	PubMed	Scopus	Web of Science
1. Spaceflight and thrombosis/coagulation	Effects of spaceflight/analogs on coagulation, platelet function, fibrinolysis and thrombosis risk	(spaceflight OR microgravity OR “spaceflight analog” OR “ground-based analog”) AND (thrombosis OR “venous thromboembolism” OR coagulation) AND (platelet OR fibrin OR fibrinolysis OR “tissue factor” OR thrombin)	7	657	15
2. Spaceflight and circadian system	Effects of spaceflight/analogs on the circadian system	(spaceflight OR microgravity OR “spaceflight analog” OR “ground-based analog”) AND (“biological clock” OR “body clock” OR “circadian clock” OR “clock gene” OR zeitgeber)	17	720	15

### 2.2 Quality assessment

Given the heterogeneity of study types, including human, animal, and *in vitro* investigations, a formal Grading of Recommendations Assessment, Development, and Evaluation analysis was not conducted. Study quality was therefore assessed qualitatively based on methodological rigor, experimental design, sample size, and the relevance of hemostasis-related outcomes.

### 2.3 Information sources and search strategy

**Figure 1:**
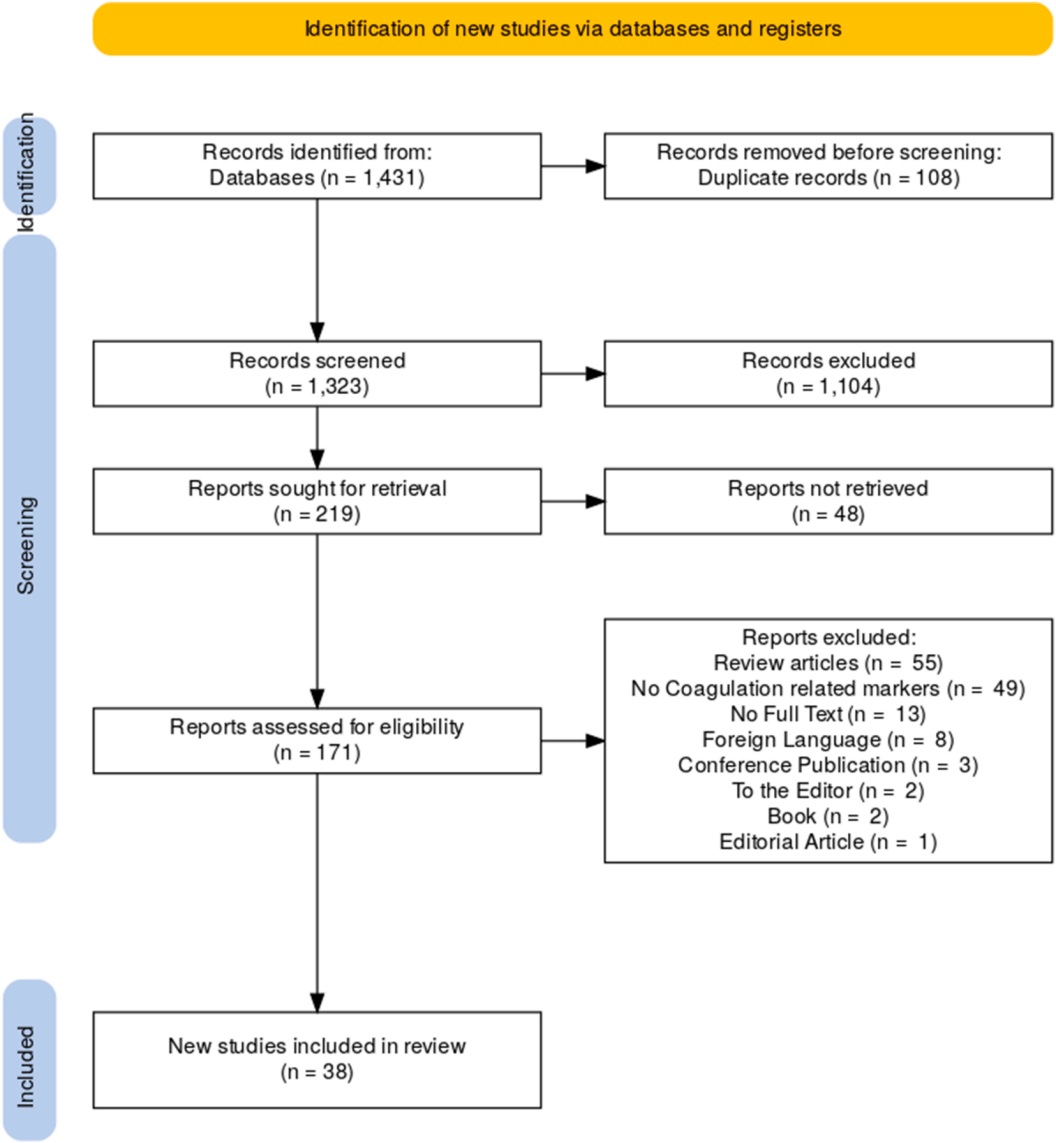
Preferred Reporting Items for Systematic Reviews and Meta-Analyses flow diagram for study selection.

A comprehensive literature search was performed across electronic databases using predefined combinations of keywords related to microgravity, spaceflight analogs, circadian rhythms, coagulation, platelet function, and thrombotic risk. The search yielded 1431 records, which were imported into the Rayyan (http://rayyan.qcri.org) website for duplicate removal as indicated in (Figure 1). Two investigators independently screened titles, abstracts, and full texts.

## 3 Results

A total of 38 studies on spaceflight and space environment models were included, of which 20 (52.6%) primarily examined hemostasis and thrombosis outcomes and 18 (47.4%) focused on circadian outcomes. No study simultaneously assessed circadian-phase regulation and hemostatic outcomes within the same experimental design. Across both domains, investigations encompassed real astronaut or cosmonaut missions, human spaceflight analogs (dry immersion and head-down bed rest), animal models, and *in vitro*–simulated microgravity or hypergravity systems.

### 3.1 Hemostasis and thrombosis in spaceflight and spaceflight analogs

Among the 20 hemostasis-focused studies (Table 3), 6 studies (30.0%) examined clinical thrombotic outcomes and risk signals in astronaut/cosmonaut cohorts or spaceflight analogs [11,12,[27], [28], [29], [30]], 3 (15.0%) performed functional platelet or coagulation assays under simulated microgravity, 9 (45.0%) applied molecular profiling approaches (proteomics, transcriptomics, miRNA, or secretome analyses), 2 (10.0%) investigated thrombopoiesis and megakaryocyte remodeling, and 4 (20.0%) evaluated nonmicrogravity space stressors, including simulated radiation and hypergravity.

**Table 3:** Overview of hemostasis-related studies in spaceflight and spaceflight analogs.

Reference	Model	Study duration/exposure	Data collection	Key findings
[27]	DI (females)	5-d DI	Multiple time points (preimmersion, during-immersion, and postimmersion)	Increased coagulability and clot firmness during immersion
[12]	Female astronauts	Short missions (≤30 d) or long (>30 d)	Preflight and postflight (no in-flight)	No overt thrombosis but altered VTE risk profile
[28]	Cosmonauts	Orbital flights of 115-205 d	Preflight and postlanding (+1 and +7)	Altered immune-hemostatic interactions and inflammatory markers
[11]	Cosmonauts	115-205 d	Preflight and early postlanding (+1 and +7)	Endothelial dysfunction and increased vascular activation markers
[29]	Cosmonauts	169-199 d	Preflight and postflight (no in-flight)	Hemorrhagic/procoagulant proteomic remodeling
[30]	DI + tilt	21 d DI	Pre-DI and day 21	Increased hemorrhagic-related protein expression
[31]	Platelet culture (RPM)	17 rpm for 5 d	postculture vs control	Delayed platelet activation under simulated microgravity
[32]	DI	3-d DI	Pre-DI, under-DI (70 h), and after-DI (+1)	Altered platelet phenotype and regulatory miRNA profiles
[33]	HU mice	20-d hindlimb unloading, followed by 14-d recovery	Sacrifice (day 21 and day 35 for recovery group)	Dysregulation of coagulation and complement pathways (multiomics)
[34]	HDBR humans	21 d of HDBR	prebed rest vs end of bed rest	Cardiovascular- and coagulation-related proteomic shifts
[35]	Astronauts	6-mo ISS missions	Preflight, in-flight, and up to ∼90 d postflight	Dynamic plasma proteome changes affecting coagulation pathways
[13]	Astronauts, and C57BL/6 male mice flown to the ISS	3 d for astronauts, for mice 35 d aboard ISS	Astronauts: pre-flight and post-flight Mice: after return day 35	Secretome alterations linked to coagulation and inflammation
[36]	Astronauts	169-199 d	Preflight and early postflight	Quantitative changes in coagulation-related proteins
[37]	Spaceflight mice	31 d ISS flight	Preflight, during flight, and postflight	Thrombotic microangiopathy and intrinsic coagulation activation
[38]	Human megakaryoblastic cell line (MEG-01) (RPM)	24, 72, 96, and 168 h	After exposure to simulated microgravity	Altered cell cycle and megakaryocyte regulation
[39]	Human UCB CD34+ progenitors → MKs/PLPs *in vitro*	(15-d differentiation), rotary-phase day 6-15	Range from day 3 to 15	Enhanced thrombopoiesis under simulated microgravity
[40]	Human endothelial cell line (HMEC-1)	Hypergravity (4 g/15 min; 20 g/15 min; and 20 g for 1-6 h)	Postexposure	Cardiovascular and endothelial remodeling
[41]	Radiation, male C57BL/6J mice	Simulated galactic cosmic radiation (simGCRsim) or *γ*-radiation (20 min)	14, 28, 365, 440, and 660 d postirradiation	Chronic thromboinflammatory cardiac remodeling
[42]	HMEC-1 human endothelial cells (*in vitro*)	Hypergravity (4 g or 20 g for 15 minutes; 20 g for 1-6 h)	Immediately postexposure	Proangiogenic and endothelial activation
[43]	Healthy volunteers	Hypergravity (long-arm centrifuge) 15 minutes at 3 Gz	Preexposure, postexposure, and 30-minute postexposure	Increased coagulation activation

DI, dry immersion; HDBR, head-down bed rest; HU, hindlimb unloading; ISS, International Space Station; MK, megakaryocytes; PLP, platelet-like particles; RPM, random positioning machine; VTE, venous thromboembolism.

#### 3.1.1 Clinical thrombotic outcomes and risk signals in spaceflight missions and analogs

Six investigations (30.0% of hemostasis studies) assessed clinically interpretable hemostatic or endothelial risk signals in dry immersion analogs and astronaut or cosmonaut cohorts [11,12,[27], [28], [29], [30]]. These studies included measurements of coagulability indices, endothelial markers, inflammatory mediators, and vascular signatures derived from clinical blood panels and proteomic analyses. Sampling was typically performed at predefined time points, including preflight, postflight, or during analog exposure.

#### 3.1.2 Functional coagulation and platelet assays under simulated microgravity

Three studies (15.0%) performed functional platelet or coagulation assays under simulated microgravity and dry immersion conditions [27,31,32]. These studies assessed platelet function, clotting parameters, and associated molecular markers, including miRNA profiles, using experimental and *in vitro* approaches.

#### 3.1.3 Molecular hemostatic signatures

Nine studies (45.0%) applied molecular profiling approaches, including proteomics, transcriptomics, miRNA profiling, or secretome analyses, across analog and flight studies [13,29,[32], [33], [34], [35], [36], [37]]. These investigations examined molecular pathways related to coagulation, endothelial function, platelet biology, and inflammatory responses.

#### 3.1.4 Thrombopoiesis and megakaryocyte remodeling

Two studies (10.0%) examined megakaryocyte differentiation and platelet production using simulated microgravity platforms, including the random positioning machine and 3-dimensional rotary cell culture systems [38,39].

#### 3.1.5 Nonmicrogravity space stressors

Four studies (20.0%) investigated space-relevant stressors independent of microgravity, including radiation exposure [40,41], and hypergravity conditions [42,43]. These studies evaluated vascular and hemostatic-related parameters under these environmental conditions.

### 3.2 Circadian regulation in spaceflight and spaceflight analogs

Among the 18 circadian-focused studies (Table 4), 10 studies (55.6%) investigated behavioral and physiological circadian outputs, including sleep architecture, temperature rhythms, and neurobehavioral performance during spaceflight analogs or confinement environments [[44], [45], [46], [47], [48], [49], [50], [51], [52], [53]]. Five studies (27.8%) examined molecular circadian mechanisms, including clock-gene expression and rhythmic transcriptomic remodeling in bed rest and rodent models [[54], [55], [56], [57], [58]]. Three studies (16.7%) evaluated autonomic circadian regulation during real spaceflight, using longitudinal chronomic analyses in astronauts [[59], [60], [61]], while 3 investigations (16.7%) explored countermeasures and Zeitgeber interventions, such as controlled lighting and exercise protocols, aimed at restoring circadian alignment under spaceflight analog conditions [44,46,52].

**Table 4:** Overview of circadian rhythm studies in spaceflight and spaceflight analog.

Reference	Model	Sampling design	Key findings
[44]	Confinement (45 d)	Repeated daily	Improved circadian entrainment with dynamic lighting
[45]	60-d HDBR	24-h phase analysis	Reduced amplitude and phase shifts in sleep rhythms
[46]	60-d HDBR	Repeated temperature	Exercise partially restores circadian phase
[47]	60-d HDBR	24-h monitoring	Circadian disruption of cardiac rhythms
[48]	60-d HDBR	Cardiac rhythm tracking	Circadian disruption of cardiac rhythms
[49]	3 d of RPM, *Drosophila* sp.	Activity tracking	Sleep and locomotor rhythm disruption
[50]	HU mice for 49 d + noise	ZT sampling	Disrupted gut microbiome circadian rhythms
[51]	3 d of DI to healthy subjects	Pre-DI, during DI, and post-DI	Multisystem circadian and physiological deconditioning
[52]	8 d of confinement, controlled lighting (healthy subjects)	Hormonal sampling	Altered endocrine circadian rhythms
[53]	21 d of HDBR humans	PSG + metabolic markers	Neural and metabolic circadian disruption
[54]	Sprague–Dawley rats + NIH3T3 cells (30° head-down tilt 28 d)	SCN tissues (at 4 h intervals over a 24 h)	NR1D1 degradation and clock disruption
[55]	90-d HDBR	4-h intervals	Global circadian transcriptome reorganization
[56]	Publicly available multiomics datasets (human and mouse skeletal muscle)	Circadian datasets: q2-4 h sampling; others: preintervention/postintervention or postflight	Clock-dependent gene dysregulation
[57]	HU male Sprague–Dawley rats; 28 d	ZT sampling	BMAL1-mediated vascular rhythm disruption
[58]	Mouse liver samples from NASA GeneLab spaceflight missions	Single end point sampling (postflight vs ground controls); STS-135 tissues collected ∼3-5 h after landing	Altered hepatic circadian metabolism
[59]	Astronauts (∼12 mo on ISS)	48-h ECG recordings at 4 phases: preflight (−156 to 157 d), early flight (18-19 d), late flight (326-327 d), and postflight (+103 to 104 d)	Altered cardiovascular circadian adaptation
[60]	Astronauts (6 men and 2 women), ISS mission (∼174.5 ± 13.8 d)	24-h ECG recordings at 5 phases: preflight (∼227 d before launch), in-flight days ∼21 (ISS01), ∼73 (ISS02), ∼156 (ISS03), and postflight (∼70 d after return)	Dynamic circadian adaptation across mission
[61]	Astronauts (8 men and 2 women), (∼171.8 ± 14.4 d on ISS)	24-h ECG recordings at 5 phases: preflight (∼234 d before launch), in-flight days ∼21 (ISS01), ∼72 (ISS02), ∼153 (ISS03), and postflight (∼77 d after return)	Altered HRV circadian structure

DI, dry immersion; ECG, electrocardiogram; HDBR, head-down bed rest; HU, hindlimb unloading; ISS, International Space Station; RPM, random positioning machine; SCN, suprachiasmatic nucleus; ZT, zeitgeber time.

#### 3.2.1 Behavioral and physiological outputs

Ten studies (55.6%) investigated behavioral and physiological circadian parameters, including sleep architecture, core body temperature, and performance measures, in spaceflight analogs and confinement environments [[44], [45], [46], [47], [48], [49], [50], [51], [52], [53]].

#### 3.2.2 Molecular clock and rhythmic transcriptomics

Five studies (27.8%) examined molecular circadian regulation using repeated sampling designs to assess clock-gene expression and transcriptomic patterns in bed rest models and animal studies [[54], [55], [56], [57], [58]].

#### 3.2.3 Autonomic rhythmicity in real microgravity

Three studies (16.7%) evaluated autonomic circadian dynamics in astronauts during spaceflight using longitudinal monitoring approaches [[59], [60], [61]].

#### 3.2.4 External synchronizers as countermeasures

Three investigations (16.7%) assessed interventions targeting circadian entrainment, including controlled lighting schedules and exercise protocols, in spaceflight analog conditions [44,46,52].

Overall, these studies examined hemostatic and circadian parameters across spaceflight and spaceflight analog environments using diverse experimental approaches.

## 4 Discussion

The most important finding of this systematic review is the absence of mechanistic studies directly linking circadian disruption to hemostatic or thrombotic outcomes in spaceflight and spaceflight analog environments. While both circadian-phase shifts, reduced rhythm amplitude, and clock-gene dysregulation and thrombotic activation of coagulation pathways and endothelial dysfunction are independently well documented, no study to date has integrated circadian phase–resolved measurements with hemostatic phenotyping within the same experimental framework.

### 4.1 Circadian–hemostatic crosstalk in the space environment

**Figure 2:**
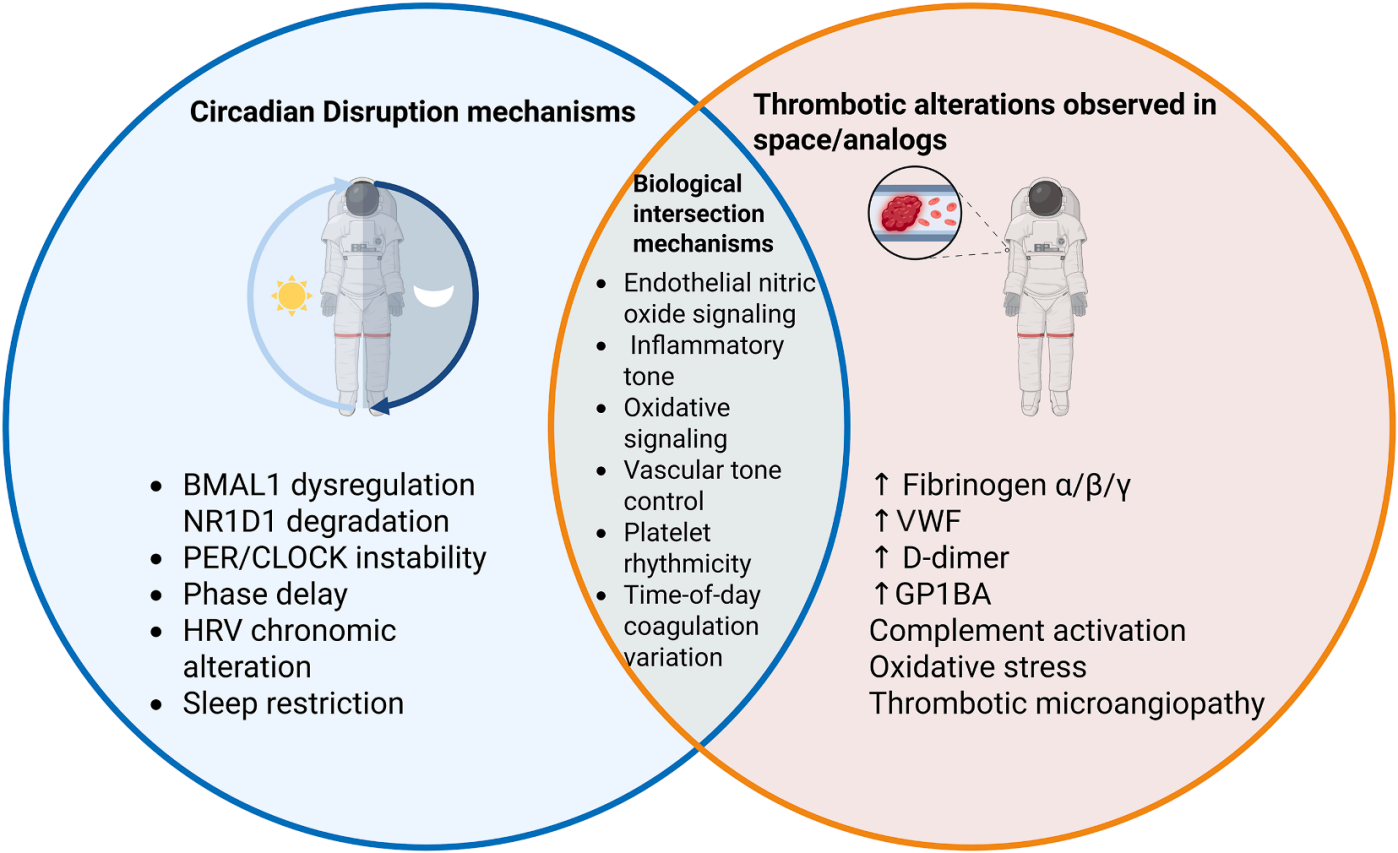
Conceptual framework linking circadian rhythm disruption to altered thrombotic susceptibility during spaceflight and spaceflight analog conditions. Exposure to the space environment disrupts circadian regulation through altered light–dark cycles, sleep restriction, confinement, and physiological stressors. These perturbations affect molecular clock networks, including dysregulation of *BMAL1*, degradation of *NR1D1* (*REV-ERBα*), and instability of *PER/CLOCK* circadian gene programs, accompanied by alterations in heart rate variability chronomics and phase delays in physiological rhythms. Concurrently, spaceflight and simulated microgravity studies report remodeling of hemostatic pathways characterized by increased fibrinogen chains, elevated von Willebrand factor (VWF), increased D-dimer, platelet glycoprotein activation (GP1BA), complement activation, oxidative stress, and thrombotic microangiopathy signatures. The overlapping region illustrates proposed circadian–hemostatic crosstalk, where circadian disruption may influence vascular tone, endothelial nitric oxide signaling, inflammatory tone, oxidative pathways, platelet rhythmicity, and time-of-day variation in coagulation activity. Together, these mechanisms suggest that thrombotic susceptibility in space may be influenced not only by gravitational and environmental stressors but also by disruption of endogenous circadian timing systems. Created with a licensed version of Biorender.com.

Thrombotic alterations during spaceflight are marked by increased fibrinogen levels, enhanced platelet activation, elevated endothelial dysfunction markers, reduced fibrinolytic activity, and increased oxidative stress signaling [12,13,27,28,30,[32], [33], [34], [35], [36], [37],[40], [41], [42], [43]]. Circadian-focused studies demonstrate clock-gene dysregulation, altered autonomic rhythmicity, disrupted metabolic oscillations, and increased inflammatory tone [[44], [45], [46], [47], [48], [49], [50], [51], [52], [53], [54], [55], [56], [57], [58], [59], [60], [61]]. Together, these observations suggest an integrated circadian–hemostatic interface in which biological timing systems may influence thrombotic susceptibility during spaceflight (Figure 2). A critical mechanistic bridge between these domains lies in core clock genes such as *BMAL1* and *NR1D1* (*REV-ERBα*). These genes form central components of the circadian timing system and regulate transcriptional programs across multiple physiological systems, including cardiovascular, metabolic, immune, and vascular pathways. Core clock transcription factors such as *BMAL1* and *CLOCK*, together with their repressors *PER* and *CRY*, generate rhythmic gene expression that extends to multiple hemostatic regulators, including clock-controlled genes such as *SERPINE1* (PAI-1), *F7*, *F12*, and *thrombomodulin* (TM; THBD), thereby linking circadian timing mechanisms to the regulation of coagulation and thrombotic susceptibility [62,63]. BMAL1 is a master transcriptional activator of circadian oscillations and directly regulates vascular tone, endothelial nitric oxide signaling, platelet reactivity, and coagulation gene expression. Experimental disruption of *BMAL1* has been shown terrestrially to impair endothelial function, increase prothrombotic gene expression, and alter diurnal variation in platelet activation. Notably, simulated microgravity studies demonstrate *BMAL1*-dependent dysregulation of vascular contractility and miR-103/CaV1.2 signaling under unloading conditions [57], indicating that gravitational stress can perturb clock-controlled vascular pathways relevant to thrombosis. However, evidence from long-duration spaceflight studies suggests that physiological responses to the space environment are not solely acute but may evolve over weeks to months as astronauts progressively adapt to microgravity [64]. Future studies should therefore determine whether such molecular and physiological changes are transient or long-lasting and whether long-duration spaceflight may be associated with partial recovery or reorganization of circadian-controlled molecular mechanisms similar to adaptations observed at the organismal level.

Similarly, *NR1D1* (*REV-ERBα*) acts as a transcriptional repressor within the circadian feedback loop and modulates inflammatory, metabolic, and hemostatic gene programs. Under simulated microgravity and isolation, mitophagy-driven degradation of *NR1D1* has been demonstrated [54], implying destabilization of circadian repression mechanisms. Given that *NR1D1* regulates inflammatory cytokines, oxidative stress responses, and metabolic pathways linked to platelet activation and fibrinogen synthesis, its disruption represents a plausible upstream regulatory pathway for the thromboinflammatory signatures observed in spaceflight proteomics studies [11,13,[28], [29], [30],[35], [36], [37]].

Despite this clear biological intersection, no thrombosis-focused study in spaceflight has directly measured clock-gene expression (eg, *BMAL1*, *NR1D1*, *CLOCK*, and *PER*) alongside coagulation end points. Conversely, circadian-focused investigations rarely quantify fibrinogen, D-dimer, thrombin–antithrombin complexes, platelet activation markers, or endothelial coagulation regulators. Thus, while molecular clock disruption and thrombotic remodeling are independently documented, their causal and mechanistic relationship in the space environment remains untested.

The hemostasis-focused literature consistently demonstrates that exposure to real or simulated spaceflight conditions induces increased coagulation activity, elevated endothelial activation markers, and altered platelet reactivity [11,12,[27], [28], [29], [30]]. A key observation across hemostasis studies is that most measurements were obtained at a single sampling time point. Consequently, reported outcomes may reflect either acute increases in coagulation markers or longer-duration adaptations in hemostatic pathways, depending on the timing of sample collection and study design. In analogs, continuous sampling throughout the intervention (eg, dry immersion) facilitates the examination of intraexposure dynamics in clot kinetics and platelet biology, increased clot firmness, accelerated clot initiation, and changes in fibrinogen and platelet indices [27] or shifts in platelet phenotype, regulatory miRNA expression, and circulating protein levels across immersion phases [32]. In contrast, numerous real-flight studies are predominantly characterized by postlanding intervals during which biomarkers such as D-dimer, von Willebrand factor, fibrinogen chains, platelet proteins (platelet factor 4/glycoprotein 1BA), endothelial markers (TM), and extensive coagulation complement are consistently elevated or activated after flight [11,13,[28], [29], [30],^35^,^36^]. However, these intervals coincide with peak occurrences of fluid shifts, plasma–volume variations, stress hormones, sleep deprivation, and external synchronizers, which can obfuscate interpretation [65,66].

In real-spaceflight and simulated microgravity, radiation, and hypergravity studies, thrombotic remodeling consistently emerges as a recurrent, yet context-dependent response to gravitational stress. Human dry immersion and head-down bed rest models demonstrate increased clot firmness, altered clot kinetics, and upregulation of fibrinogen- and platelet-related pathways [27,30,32,34]. Multiomics analyses in hindlimb-unloading mice further identified suppression of C1 inhibitor and increased thrombospondin-1 expression, alongside activation of complement and coagulation cascades [33]. *In vitro* megakaryocyte and platelet systems revealed gravity-sensitive alterations in thrombopoiesis, cytoskeletal organization, and ion channel expression [31,38,39], suggesting that microgravity influences both platelet production and activation dynamics.

In real spaceflight, proteomic investigations consistently report transient increases in coagulation factor expression and platelet-associated pathway activation during early readaptation to Earth gravity [11,13,[28], [29], [30],^35^,^36^]. Elevations in fibrinogen chains, platelet glycoproteins (eg, glycoprotein 1BA), von Willebrand factor, D-dimer, factor XI, and complement components were observed after flight [11,13,[28], [29], [30],^35^,^36^]. Nrf2-deficient murine spaceflight models demonstrated exacerbated thrombotic microangiopathy signatures and upregulation of intrinsic coagulation genes, including fibrinogen α/β/γ chains and contact pathway components [37]. However, overt clinical thrombosis remained rare, and hematologic indices generally remained within terrestrial venous thromboembolism risk thresholds [12].

Space-radiation models revealed chronic thromboinflammatory activation characterized by increased complement activation, extracellular matrix deposition, neutrophil extracellular trap formation, and reduced systolic function [40,41]. In contrast, exposure to hypergravity induces acute hypercoagulability with shortened clotting times, increased fibrinogen, elevated thrombin–antithrombin complexes, and enhanced platelet activation [42,43].

Importantly, despite frequent reporting of mission duration, recovery phase timing, and controlled housing light–dark cycles in animal experiments, none of the thrombosis-focused studies incorporated circadian phase–resolved sampling or time-of-day stratification of hemostatic endpoints. Most sampling was anchored to operational milestones (eg, landing day +1 or +7 and immersion day 21), rather than biological time. Thus, although coagulation remodeling is evident, its interaction with endogenous circadian regulation remains untested in spaceflight contexts.

The circadian literature is methodologically designed to interrogate temporal organization. Bed rest and confinement studies used 24- to 48-hour sampling windows, repeated 4-hour collections, and continuous physiological monitoring [[44], [45], [46], [47], [48],^55^]. Dynamic lighting schedules and confinement studies demonstrated delayed circadian-phase timing and reduced rhythm amplitude of core body temperature and performance rhythms. Simulated microgravity influenced sleep architecture and neural-metabolic coupling, as demonstrated by disrupted sleep onset patterns and altered resting-state neural networks [53].

Animal and cellular studies further revealed molecular clock disruption under simulated microgravity. Mitophagy-driven degradation of *NR1D1* (*REV-ERBα*) was demonstrated, indicating reduced stability of circadian repression pathways [54]. Transcriptomic analyses revealed downregulation and phase misalignment of core clock genes (*BMAL1*, *CLOCK*, *PER*, and *CRY*), which coordinate downstream metabolic and physiological rhythms [55]. Spaceflight rodent studies identified clock-dependent dysregulation of skeletal muscle genes [56] and liver lipid metabolism across missions [58]. Vascular *BMAL1*-dependent signaling was disrupted under simulated microgravity, altering diurnal contractility patterns [57].

Long-duration astronaut investigations showed altered heart rate variability time structure, reduced rhythmic stability, and phase-dependent autonomic shifts, indicating that circadian responses evolve dynamically across mission duration [[59], [60], [61]], emphasizing that circadian responses in space show progressive phase shifts and reduced rhythmic stability across mission duration rather than static. Moreover, Otsuka et al. [61] reported that heart rate variability analyses in astronauts during long-duration ISS missions suggest that coupling between 12- and 24-hour rhythms may support physiological adaptation to the space environment, while background fluctuations in magnetic fields may further modulate circadian adaptation processes [61].

### 4.2 Critical knowledge gaps

**Figure 3:**
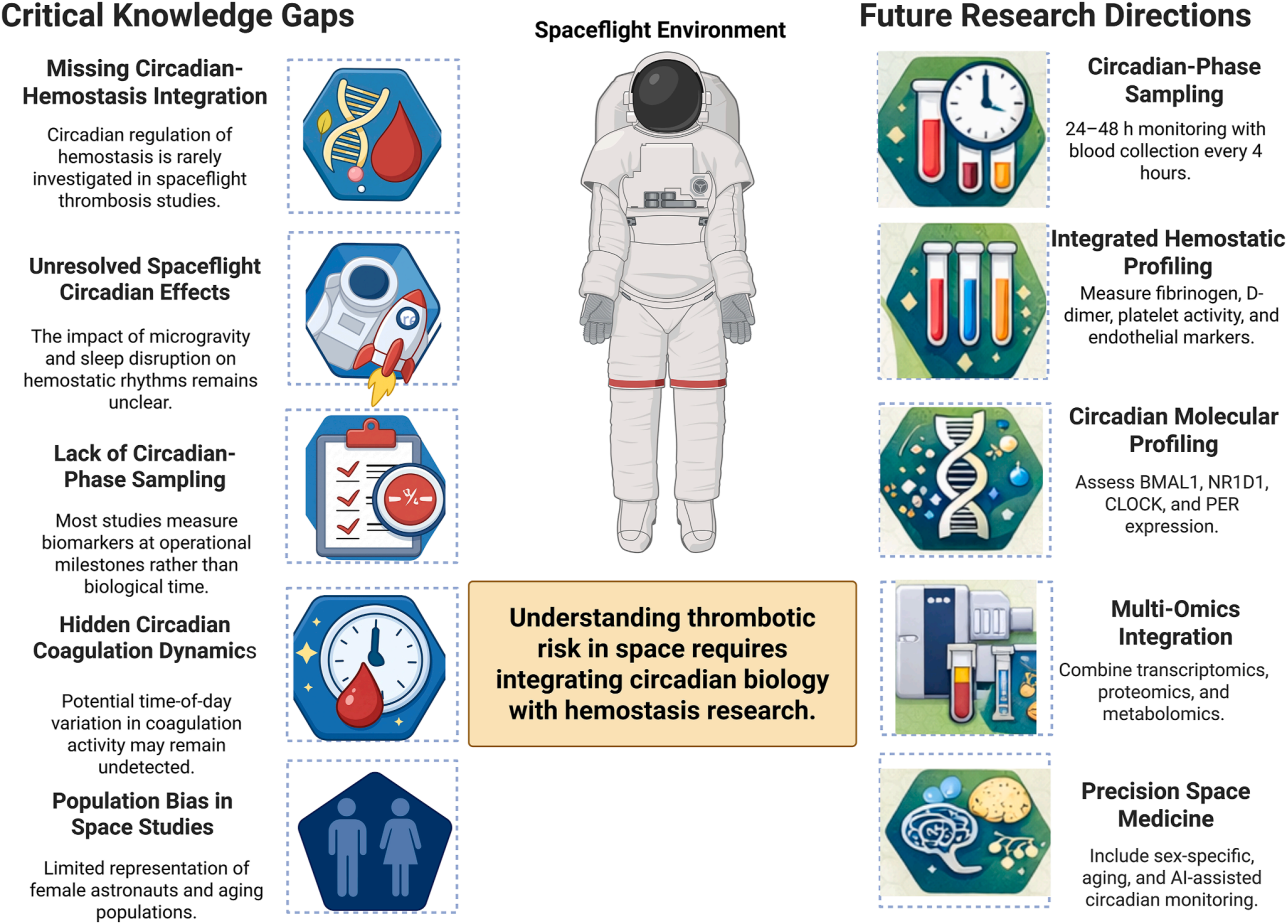
Conceptual overview of critical knowledge gaps and future research directions linking circadian biology and hemostasis in the space environment. The left panel summarizes key limitations in current spaceflight and analog studies, including the lack of circadian-phase sampling, unresolved circadian effects on hemostatic regulation, and limited representation of sex-specific responses. The right panel highlights proposed research strategies, including circadian phase–resolved blood sampling, integrated hemostatic profiling, molecular clock-gene measurements, multiomics integration, and precision space medicine approaches. Together, these approaches aim to clarify whether thrombotic susceptibility during spaceflight is influenced not only by gravitational and environmental stressors but also by disrupted biological timing. Created with a licensed version of Biorender.com.

Terrestrial chronobiology studies consistently demonstrate that circadian misalignment increases thrombotic susceptibility through clock-gene dysregulation, altered platelet rhythmicity, endothelial dysfunction, and pronounced morning surges in coagulation activity. In controlled human and animal studies on Earth, time-of-day–resolved sampling has revealed rhythmic variation in platelet activation, fibrinolytic balance, and coagulation factors, supporting the concept that hemostasis is tightly regulated by the circadian timing system. In the spaceflight environment, however, multiple factors, including unstable light–dark exposure, mission-phase stress, sleep restriction, and cephalad fluid redistribution may disrupt these same clock-controlled hemostatic networks (Figure 3). Despite this plausible biological link, most spaceflight and analog thrombosis investigations rely on sampling anchored to operational milestones (eg, preflight, landing day, or recovery days) rather than circadian phase. Consequently, potential time-of-day–dependent oscillations in coagulation activity may remain undetected in current datasets. Direct in-flight circadian-phase measurements of hemostatic parameters remain rare, and interpretations are therefore largely inferred from terrestrial chronobiology and analog studies. Emerging digital medicine approaches, including continuous physiological monitoring and artificial intelligence–based analysis of sleep and circadian biomarkers, may help resolve these dynamics in future missions. Complementary experimental platforms such as microgravity-compatible organ-on-a-chip systems may also help investigate circadian–hemostatic interactions under controlled conditions when continuous astronaut sampling is not feasible. Additionally, most astronaut cohorts and space analog studies have historically been male dominated, and sex-specific analyses of circadian or hemostatic responses remain limited, representing an important knowledge gap given known hormonal and coagulation differences between sexes.

### 4.3 Limitations and future directions

The need for targeted investigation of thrombotic mechanisms in spaceflight has also been emphasized by international expert groups, including the European Space Agency Venous Thrombosis Topical Team, which highlighted major gaps in understanding thrombosis risk, diagnosis, and prevention strategies in the space environment [16]. Future investigations should integrate circadian phase–resolved sampling frameworks with hemostatic phenotyping to directly test the interaction between biological timing and thrombotic regulation in spaceflight environments. Multiday 4-hour interval blood collection during analog exposure or defined mission phases should incorporate clock-gene expression (*BMAL1*, *NR1D1*, *CLOCK*, and *PER*), fibrinogen levels, D-dimer, thrombin–antithrombin complexes, platelet function assays, endothelial markers (von Willebrand factor and TM), and complement components (Figure 3). Only such chronothrombotic designs can determine whether thrombotic remodeling in space is partially driven by disrupted biological timing rather than solely by mechanical unloading, radiation exposure, or fluid shifts. Integration with transcriptomics, proteomics, and metabolomics data sets will further clarify clock-controlled thrombotic pathways. Future studies should also incorporate sex-specific analyses, as hormonal regulation, platelet reactivity, and coagulation pathways differ between men and women, yet female-focused investigations remain limited in spaceflight and analog research. Moreover, as future space flights are likely to include older individuals with an increased thrombotic risk, the impact of aging at the interface between hemostasis and circadian disruption must be considered.

In addition, the assessment of thrombotic risk in spaceflight is limited by the small number of individuals and the rarity of clinically overt events, making traditional event-based analyses challenging. Future studies should therefore adopt longitudinal, within-subject designs integrating vascular imaging with coagulation, platelet, and endothelial biomarkers to detect subclinical changes in thrombotic pathways. However, the feasibility of performing such assays during spaceflight remains an important challenge, as platelet function testing requires fresh samples, rapid processing, and controlled preanalytical conditions that may be difficult to maintain in microgravity. Although blood collection and plasma separation are feasible onboard, microgravity may affect fluid handling and sample processing, potentially introducing variability in assay results [1]. The inclusion of commercial spaceflight participants may help expand sample size and population diversity; however, differences in baseline characteristics and mission profiles must be carefully considered [1,67]. Despite these opportunities, challenges such as limited in-flight sampling and interindividual variability remain, highlighting the need for standardized protocols and collaborative data-sharing efforts. The major knowledge gaps and future research priorities emerging from the literature are summarized in Figure 3, which illustrates the need for integrating circadian biology with hemostatic profiling in spaceflight investigations.

## 5 Conclusion

Despite extensive evidence for both circadian disruption and hemostatic remodeling in spaceflight, their mechanistic interaction remains unexplored. Addressing this gap through circadian phase–resolved, multiomics, and functional hemostatic studies will be essential to determine whether disrupted biological timing is a causal driver of thrombotic risk during long-duration missions.
